# Seasonal population dynamics of *Sargassum fusiforme* (Fucales, Phaeophyta), Suo-Oshima Is., Seto Inland Sea, Japan—development processes of a stand characterized by high density and productivity

**DOI:** 10.1007/s10811-016-0951-z

**Published:** 2016-09-09

**Authors:** Goro Yoshida, Hiromori Shimabukuro

**Affiliations:** Japan Fisheries Research and Education Agency, National Research Institute of Fisheries and Environment of Inland Sea, Maruishi 2-17-5, Hatsukaichi, 739-0452 Hiroshima Japan

**Keywords:** Biomass, Density, Population structure, Productivity, *Sargassum fusiforme*

## Abstract

Seasonal population dynamics of *Sargassum fusiforme*, one of the most important edible macroalgae in Japan, were studied. Recruits were mainly generated by vegetative reproduction at the margins of filamentous holdfasts. They first appeared in late spring and peaked in summer as upright thalli of the previous generation withered. After producing recruits, holdfasts withered indicating that holdfasts were also annual, the same as upright thalli. All recruits produced main branches and became new upright thalli in early autumn. During this transitional period, the thallus density decreased due to the crowded conditions induced by simultaneous growth initiation. After this early mortality, however, thallus density remained almost constant over much of the growth season. Thallus growth continued during winter and the stand biomass peaked in spring. During this biomass accumulation, development of a thallus size hierarchy was moderate and no size-dependent mortality was observed. Main branch number per thallus was also constant until spring, indicating the main branches also persisted after being produced in early autumn. This lack of severe intraspecific competition both at a thallus and main branch level is supported by the ambient wave condition of the habitat which gives moderate undulation and enables light and nutrients to be supplied to each thallus, and allowed the *S. fusiforme* stand to maintain its densely-packed feature with a high productivity.

## Introduction

*Sargassum fusiforme* (Harvey) Setchell, called *hijiki* in Japan, is an edible macroalga with a distribution around the East Asian coasts including Japan, Korea, and China (Yoshida 1998). Although *hijiki* is a traditional food in Japan, recent interest of consumers for a healthy diet has led to an increase in its consumption reaching 12,000–15,000 t dry weight (D.W.) per year. However, over 90 % of the consumption depends on products imported from China and Korea (Ofusa 2011). Demand for domestic *hijiki* has been increasing by both the seaweed processing distribution industry and consumers of Japan (Ito et al. 2008, 2009b).

Until recently, production of *S. fusiforme* in Japan mostly relied on natural resources (Ito et al. 2008), in contrast to the large-scale aquaculture production in China and Korea (Hwang et al. 1999; Pang et al. 2005, 2006). Aquaculture of *S. fusiforme* in Japan remains limited due to problems in relation to mass seeding production for culture and high labor costs. Because of the increasing demand for domestic *hijiki* in Japan, there are concerns about over-harvesting in the *hijiki* fishery causing damage to local resources. For sustainable production of *S. fusiforme*, establishment of managed harvest based on an understanding of the ecological characteristics of the species is essential.

**Fig. 1:**
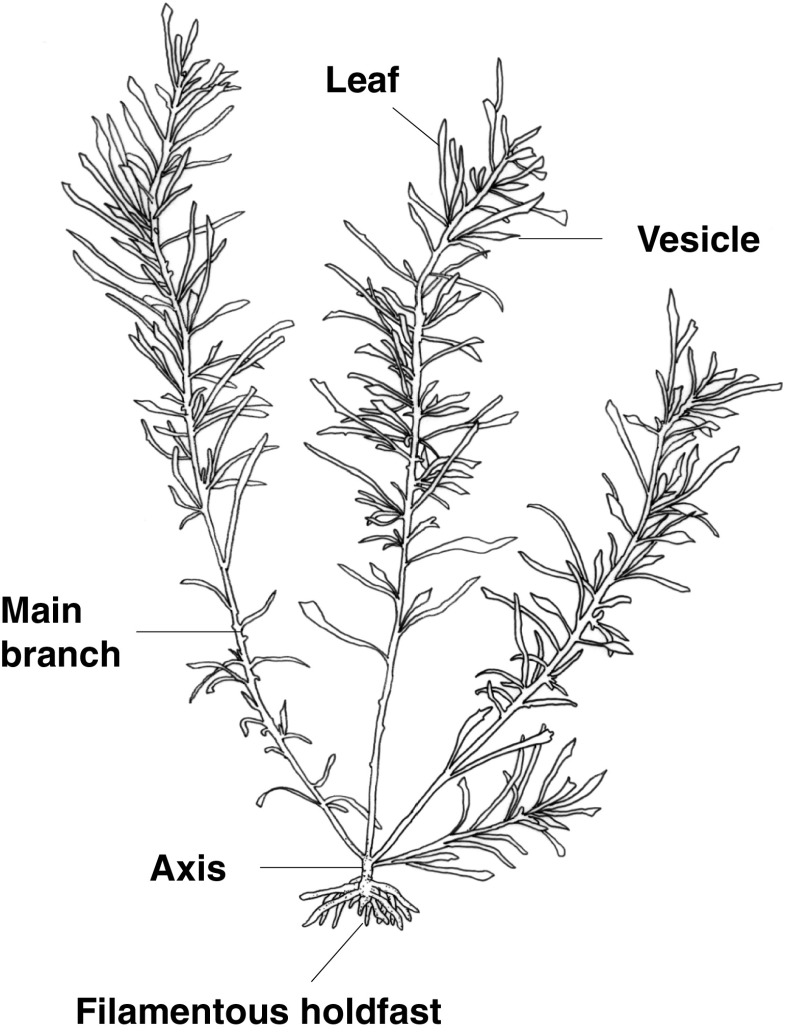
Drawing of an upright thallus and filamentous holdfast of *Sargassum fusiforme.* Parts of a thallus which were counted (main branches) or measured for their biomass are also shown.

*Sargassum fusiforme* makes stands in intertidal zones of rocky shores and attaches to the hard substrata by filamentous holdfasts formed at the base of the upright thallus (Fig. 1, Arai 1993; Yoshida 1998; Shimabukuro et al. 2016). The upright thallus produces several main branches, also referred to as primary laterals or “modules” (e.g., Largo and Ohno 1992; Andrew and Viejo 1998; Ateweberhan et al. 2005, 2008), at the top of its short axis (Fig. 1), and the development of the stands is due to the growth of the main branches from each thallus (Arai 1993; Yoshida 1998; Shimabukuro et al. 2016). It has been reported that populations of *S. fusiforme* are mainly maintained by vegetative reproduction, in which recruits are produced at the margins of filamentous holdfasts (Arai and Arai 1983), although recruits originating from sexual reproduction also have the ability to form stands (Yotsui et al. 1996).

Several ecological studies on *S. fusiforme* have been done in Japan, concerning its phenology in growth and reproduction (e.g., Terawaki 1985; Suwa 2014), as well as biomass and thallus density in the harvest season (e.g., Katada 1940; Suwa 2014). In these studies, the characteristics of high-density stands of *S. fusiforme* have been shown, and in one of the oldest studies, a positive relationship between thallus density and biomass in the harvest season was reported (Katada 1940). This is considered to be unlikely to occur in large brown algae where intraspecific competition or “self-thinning,” which is a density-dependent mortality process in the course of population development, is common (e.g., Creed et al. 1998; Arenas and Fernández 2000; Rivera and Scrosati 2008). Whether these intrinsic self-regulating events which could affect harvest output actually occur in *S. fusiforme* populations is, however, unknown because of the lack of information on the temporal dynamics of its population structure.

In this study, we monitored and described the seasonal changes in the structure of a *S. fusiforme* population in the Seto Inland Sea, Japan. We found a unique feature in the temporal population dynamics, that is, a lack of severe intraspecific competition in the seasonal development process of the population. The lack of severe intraspecific competition allowed the *S. fusiforme* population studied to maintain a high thallus density and exhibit high productivity at its seasonal peak.

## Materials and methods

### Study site

**Fig. 2:**
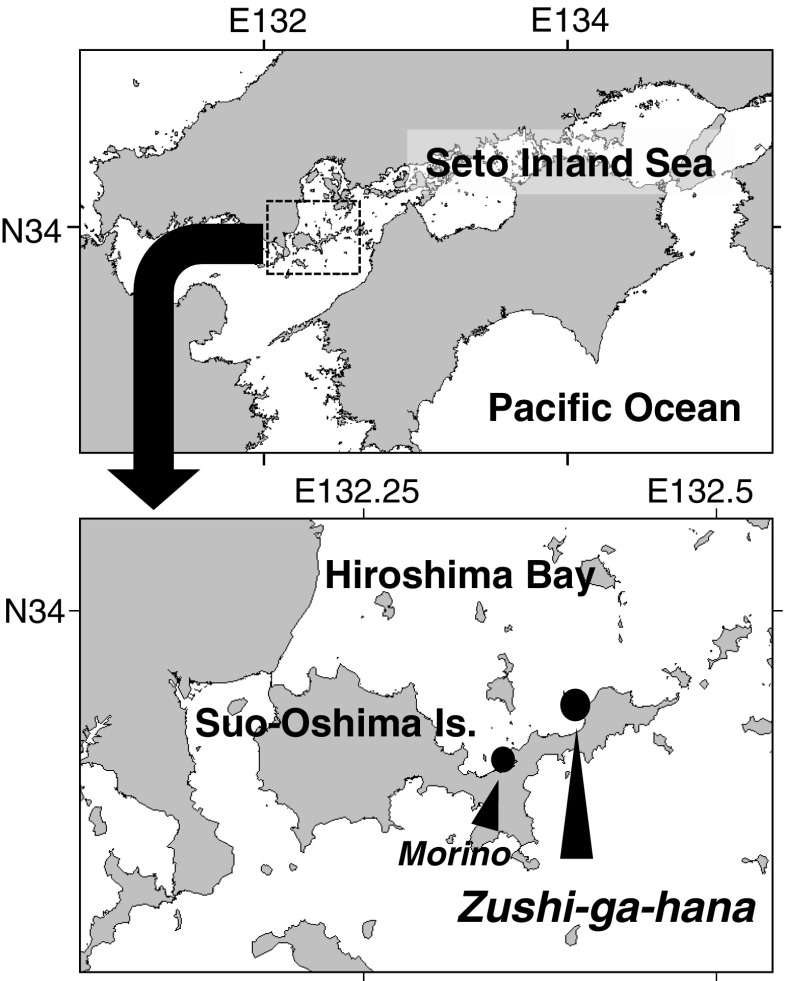
Map showing the study site, Zushi-ga-hana, on the northern coast of Suo-Oshima Is. in Hiroshima Bay, western Seto Inland Sea, Japan. Morino which is the monitoring site of water temperature is also shown

This study was conducted at Zushi-ga-hana (N 33.94, E 132.40), on the northern coast of Suo-Oshima Is. facing Hiroshima Bay in the western Seto Inland Sea (Fig. 2). At Zushi-ga-hana, a rocky reef is exposed on a shallow sandy sea bottom and provides a substratum for macroalgae from the intertidal to 1 m depth (in Chart Datum Level). *S. fusiforme* forms a dense monospecific stand in the intertidal zone of the reef (+0.4~+1.4 m), whereas *Sargassum horneri* (Turner) C. Agardh and *Sargassum patens* C. Agardh are dominant in the subtidal zone of the reef, just below the *S. fusiforme* zone. During the spring tides, the sea level fluctuates over 3 m a day at the maximum. Zushi-ga-hana is not a fishery ground of *hijiki* for local fishermen, so there was no harvest during the study period.

**Fig. 3:**
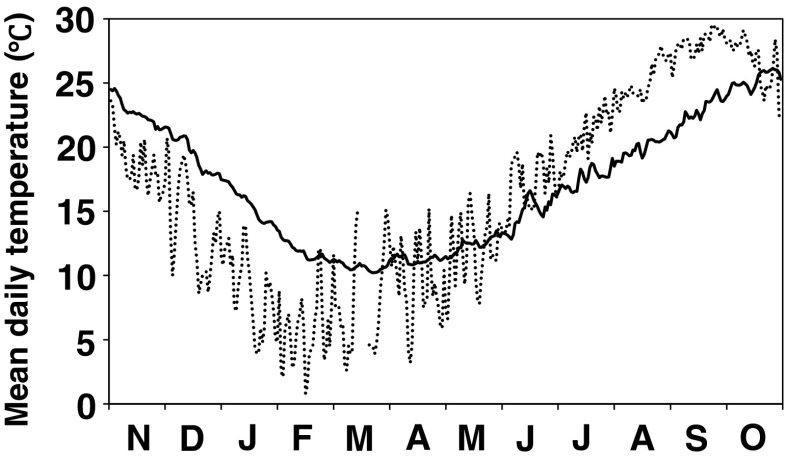
Fluctuation of daily mean seawater temperature (*solid line*) monitored at 5-m depth of Morino which is 6 km apart from the study site. Daily mean air temperature (*dotted line*), which was monitored at the Age-no-sho Meteorological Observatory in Suo-Oshima Is. (Japan Meteorological Agency 2016), is also shown

Mean daily water temperature during this study ranged from 10.2 °C in mid February to 26.1 °C in mid September, which was monitored by setting TidbiT v2 Temp Loggers (Onset) in the subtidal zone (5-m depth) at Morino, ca. 6 km apart from Zushi-ga-hana (Figs 2, 3). In an additional monitoring in summer at Zushi-ga-hana with the same way as at Morino, the temperature of ambient water of *S. fusiforme* zone was 0–2.4 °C higher than values monitored at Morino due to water stratification in summer (Fig. 3). Mean daily air temperature from the Age-no-sho Meteorological Observatory in Suo-Oshima Is. during the study also ranged from 0.9 °C in January to 33.6 °C in August (Fig. 3), with an instantaneous minimum of −2.2 °C and maximum of 35.6 °C (Japan Meteorological Agency 2016).

### Sampling, sample processing, and population structure analysis

Sampling was conducted monthly from November 2009 to October 2010. Five or six quadrats of 0.04 m^2^ were placed randomly in the *S. fusiforme* stand, and upright thalli with filamentous holdfasts were carefully collected using scrapers. Care was taken for holdfast sampling to avoid missing any residual holdfasts on the rock. Only in August when numerous recruits without branches appeared in high densities, 0.01 m^2^ quadrats were used instead of 0.04 m^2^ quadrats to reduce the excess sampling requirement. The samples from each quadrat were put into separate meshbags and brought back to the laboratory of National Research Institute of Fisheries and Environment of Inland Sea (Maruishi, Hatsukaichi City, Hiroshima Prefecture).

In the laboratory, upright thalli and recruits without main branches were separated from filamentous holdfasts and counted for thallus density estimation. For thalli with main branches, total length, which was the length from the base to the apex of thallus, and fresh weight were measured and the number of main branches were counted for all thalli sampled except for the September sample. For the September sample, length and weight were measured for 100 thalli randomly selected, but a count of the number of main branches was not conducted. For recruits without main branches, length was measured for randomly selected thalli (*n* = 30), and fresh weight per a recruit was estimated as biomass/density. In this case, it was difficult to discriminate between recruits vegetatively reproduced from filamentous holdfasts (ramets) and recruits that developed from propagules (genets), because they follow identical morphological development processes (Arai 1993).

After measurement, thalli were pooled for each quadrat and dried at 85 °C for a few days. The dried thalli were separated into either (1) axis and branches including both the main and lateral branches or (2) leaves and vesicles which are similar in their morphologies (Arai 1993; Yoshida 1998; Shimabukuro et al. 2016), and in their sexual reproduction season or (3) receptacles. After separating out the parts, biomass of each was weighed. Filamentous holdfasts were also dried and weighed for each quadrat after cleaning off any adhesive matter, such as tubes of polychaetes and barnacles.

After March, growth of epibionts, mainly hydrozoans, became conspicuous on upright thalli which could have caused an overestimation of biomass. The biomass of these epibionts were examined for two quadrats in March and June and estimated to be 6.8 and 30.0 % of the total weight of dried samples. For samples in April and May, the proportion of the epibionts in the total biomass was assumed to be 18.4 %, which was simply the mean of the proportions in March and June. Biomass of *S. fusiforme* was estimated by subtracting these portions of epibionts from the total biomass.

Seasonal dynamics of the population structure was described not only as changes in density, biomass, and mean thallus size but also as transition in size hierarchy of the thalli in the population. As an index representing the size hierarchy in populations, the Gini coefficient is a statistic frequently used in the analysis of plant population structures including many cases of macroalgae (e.g., Ang and DeWreede 1992; Santos 1995; Creed et al. 1998; Arenas and Fernández 2000; Rivera and Scrosati 2008). It accurately reflects the size inequality among thalli in the population (Weiner and Solbrig 1984), ranging from a minimum value of 0 (perfect equality of plant size in the population) to a theoretically maximum value 1 (perfect inequality) (Rivera and Scrosati 2008). The Gini coefficient for thallus length was calculated for each quadrat sample except for the July and August samples. Total sample size of the July sample was small, so thalli of all quadrats were pooled for the calculation. In the case of the August sample in which all thalli were recruits, only a portion of thalli were measured in length, so the data of all thalli measured were used for the calculation. Calculation of the Gini coefficient was carried out with a package of “ineq” in the statistical software R version 3.2.2 (Zeileis 2014).

### Statistical analyses

Significance in seasonal variations of all parameters of the population was examined using the Kruskal–Wallis test, instead of parametric one-way ANOVA, as most of our data did not meet the assumptions of homogeneity of variances as well as normal distribution which are required for conducting parametric ANOVA. The homogeneity of variances and normality was checked by Levene’s test and Shapiro–Wilk test, respectively. After significant seasonal differences were detected by Kruskal–Wallis test, a Games–Howell test was conducted for multiple comparisons. All these statistical analyses were conducted using SPSS 20.0 Statistic (IBM).

## Results

### Seasonal population development

When the research was begun in November 2009, all thalli of *S. fusiforme* had already developed several main branches. Thallus growth continued in winter until spring when it reached its peak. Sexual maturation, which was shown by receptacle formation, and subsequent senescence of thalli were observed in June and July. All of the upright thalli of the “2009 cohort” completely disappeared by August. Recruits of the 2010 cohort were first observed in May 2010 and increased during the summer. After September, recruits began to elongate main branches and developed into upright thalli.

Though we observed only partial seasonal fluctuation of each of the two cohorts in 1 year, every parameter of the population exhibited a smooth seasonal transition. Therefore, it was assumed that the seasonal transitions observed represent a typical fluctuation of an identical cohort of *S. fusiforme* at the study site.

### Seasonal changes in thallus density

**Fig. 4:**
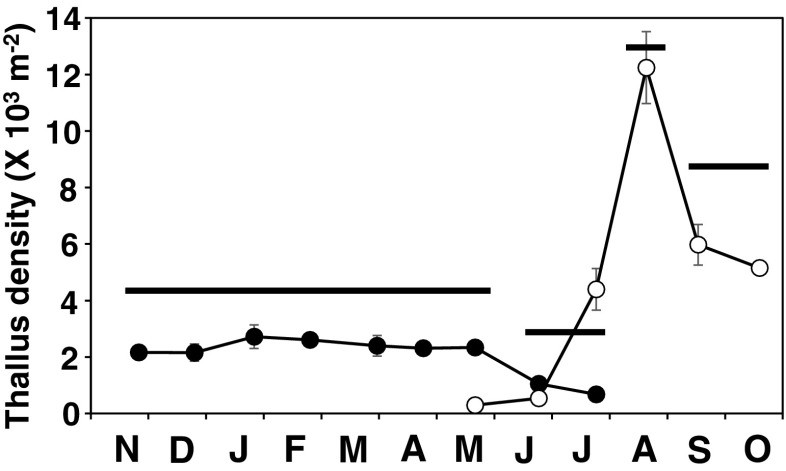
Changes in *S. fusiforme* thallus density of the 2009 (*filled circles*) and 2010 (*empty circles*) cohorts estimated by quadrat samplings (*n* = 5 or 6). *Error bars* indicate standard errors. Games–Howell test was conducted for the 2009 cohort and the 2010 cohort after August and *horizontal bars* indicate the results. *Bars at the same level* are not significantly different (*p* > 0.05)

Seasonal fluctuations in mean thallus density (Fig. 4) were significant in Kruskal–Wallis test (*p* < 0.001). However, mean thallus density of the 2009 cohort was almost constant around 2500 thalli m^−2^ from November to May 2010 and there was no significant difference during that period (Games–Howell test, *p* > 0.05). The decrease in density from May to July was significant (*p* < 0.05).

Recruits first observed in May reached the maximum density in August (>12,000 thalli m^−2^). In September, when recruits began to form and elongate main branches, the density decreased significantly to below 6000 thalli m^−2^ (*p* < 0.05).

### Seasonal changes in thallus size

**Fig. 5:**
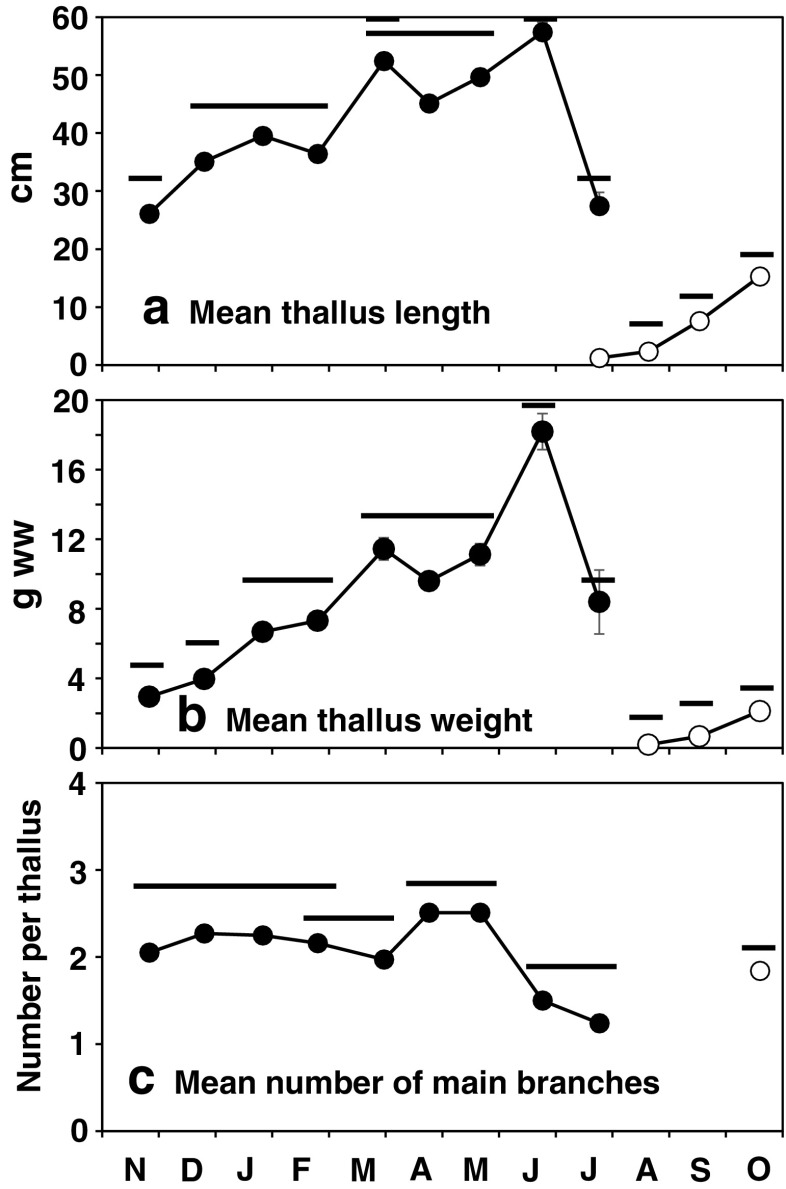
Changes in mean thallus length (**a**), weight (**b**), and number of main branches (**c**) of *S. fusiforme* thalli of the 2009 (*filled circles*) and 2010 (*empty circles*) cohorts. *Error bars* indicate standard errors. Games–Howell test was conducted for the 2009 cohort and the 2010 cohort after August and *horizontal bars* indicate the results. *Bars* at the same level are not significantly different (*p* > 0.05)

Growth of upright thalli began with the elongation of main branches in autumn, which was shown in thallus length increase of the 2010 cohort after September (Fig. 5a). Assuming the 2010 cohort followed the same trajectory as the 2009 cohort, the growth continued during winter and spring. Mean thallus length reached the maximum 57.4 cm in June, though there were stagnation periods (January to February, and March to April). The seasonal transition in thallus length was significant (Kruskal–Wallis test, *p* < 0.001). Thallus fresh weight also fluctuated significantly (Kruskal–Wallis test, *p* < 0.001) (Fig. 5b). Thallus weight in March to June was probably overestimated because it was very difficult to remove epibionts from fresh thalli.

**Fig. 6:**
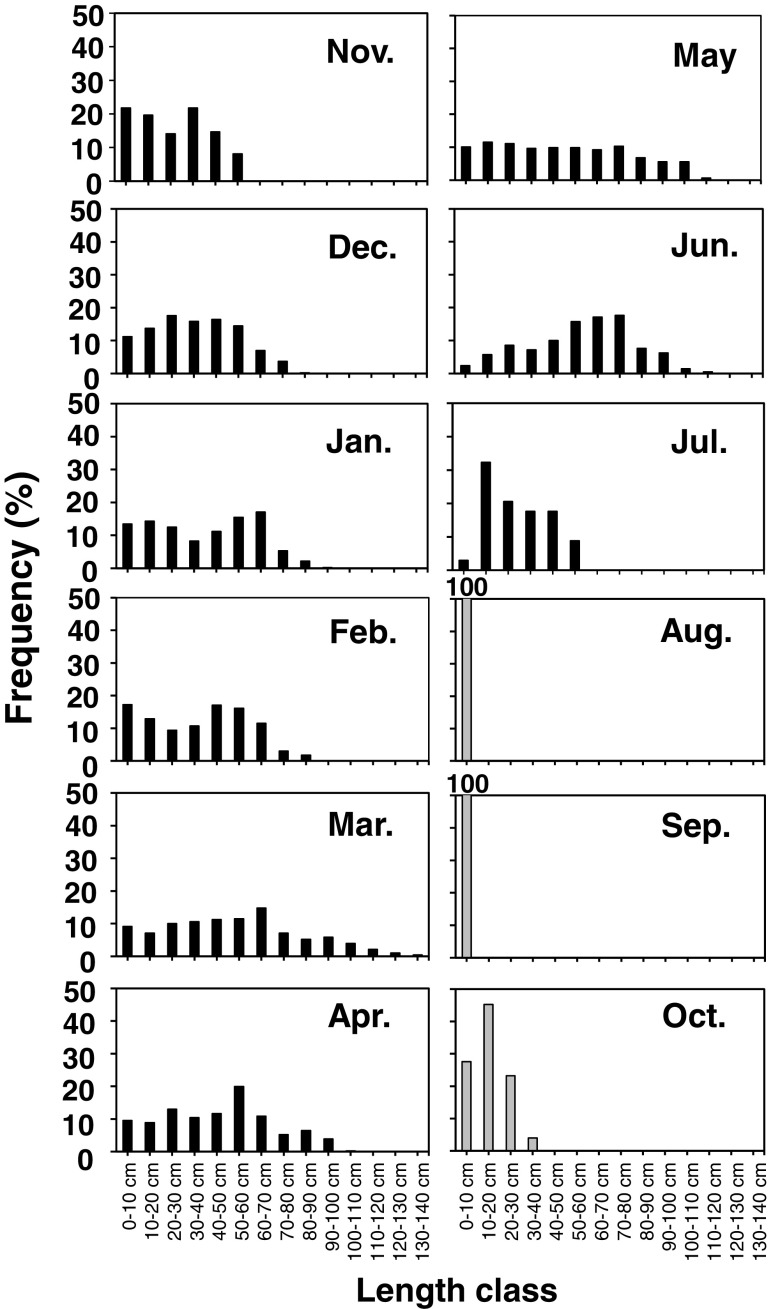
Size class-frequency distributions of *S. fusiforme* thalli of the 2009 cohort (indicated by *black bars*) and the 2010 cohort after August (indicated by *gray bars*)

In Fig. 6, thallus length frequency distributions of the 2009 and the 2010 cohort (only after August) is shown. For the 2009 cohort, the mode of the distribution was unclear for most of the study period. Some thalli grew to over 100 cm in length in March and May, but they accounted for only small portion. Smaller thalli exhibited a relatively even distribution in all length classes up to May. For the 2010 cohort, all young thalli (recruits) belonged to the smallest class until September. Differentiation in size among thalli developed in October after the main branches began to elongate (Fig. 6).

### Number of main branches per thallus

**Fig. 7:**
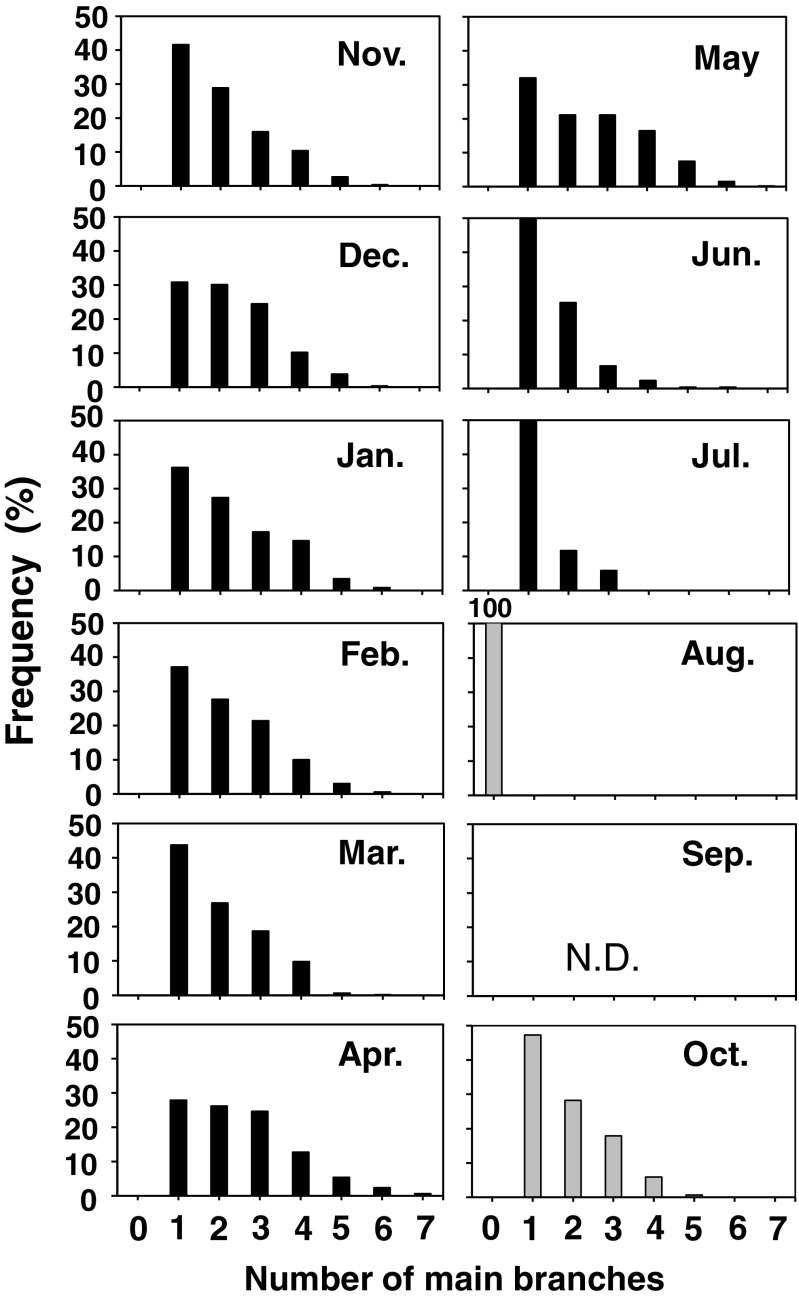
Frequency distributions of the number of main branches of *S. fusiforme* thalli of the 2009 cohort (indicated by *black bars*) and the 2010 cohort after August (indicated by *gray bars*)

The mean number of main branches per thallus (Fig. 5c) for the 2009 cohort was relatively constant (2–2.5 branches per one thallus) during most of the growth season, though a slight decrease in March was statistically significant (Games–Howell test, *p* < 0.05). Also, the frequency of the number of main branches of thalli in the population did not exhibited dramatic changes during these seasons (Fig. 7). Thalli, which had five or more branches, were only a few in number. For the 2010 cohort, though the frequency in main branch number of the September sample was not evaluated, many thalli were observed to have already produced two or three branches. In October, there were no thalli without main branches and several thalli already had five branches. The distribution in the frequency of number of main branches in October (for the 2010 cohort) was already similar with those after November (for the 2009 cohort, Fig. 7).

**Fig. 8:**
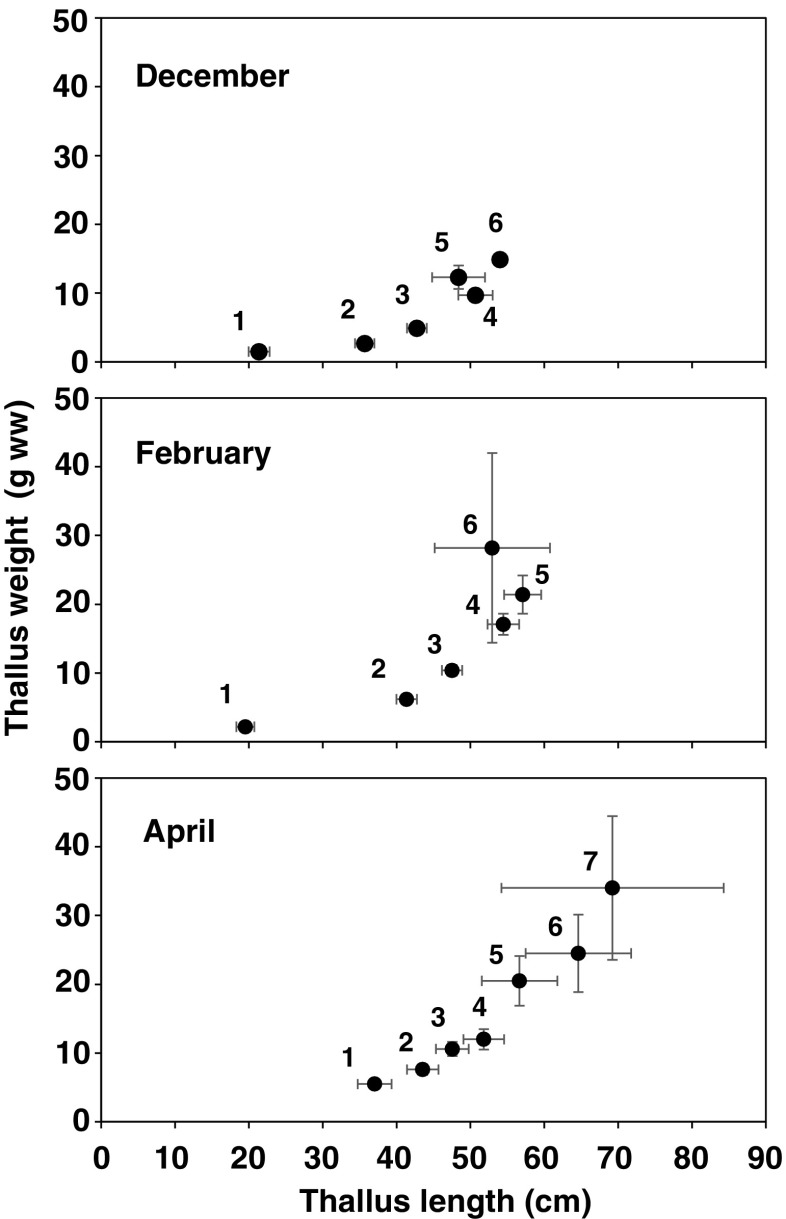
Relationship between thallus length and weight of *S. fusiforme* thalli with different numbers of main branches. *Numbers* indicate the number of main branches the thalli possessed. *Error bars* indicate standard errors

In Fig. 8, relationship between the main branch number per one thallus and the thallus size for the December, February, and April samples are shown. The standard errors were larger in thalli with larger numbers of branches due to their small sample size. In each month, thalli which had produced more branches exhibited larger thallus length and weight than thalli with fewer branches. The difference in length and weight among the thalli with different branch numbers was statistically significant (Kruskal–Wallis test, *p* < 0.001) in each month. Also, length and weight of thalli with identical number of branches was significantly different among different months for thalli with 1–3 branches (Kruskal–Wallis test, *p* < 0.05), indicating growth of those thalli. For thalli with 4–5 branches, only thallus weight was significantly different among the different months (Kruskal–Wallis test, *p* < 0.05).

### Biomass

**Fig. 9:**
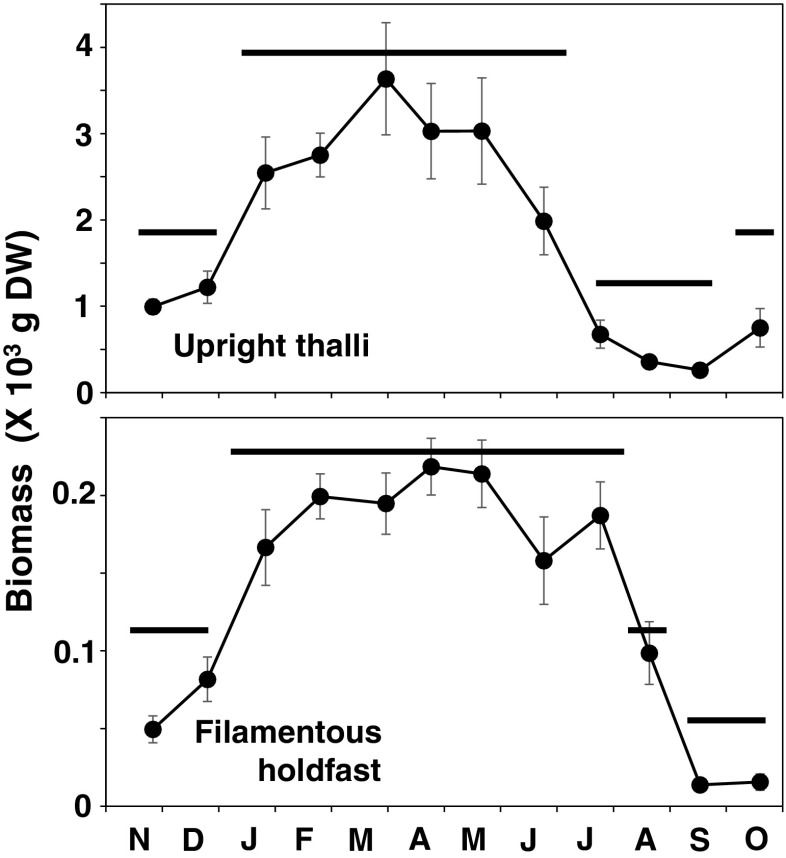
Changes in biomass of thalli and filamentous holdfasts of *S. fusiforme* by quadrat samplings (*n* = 5 or 6). Biomass of the 2009 and 2010 cohorts was pooled. *Error bars* indicate standard errors. *Horizontal bars* indicate the result of Games–Howell test and bars at the same level are not significantly different (*p* > 0.05)

Seasonal changes in biomass for both the 2009 and the 2010 cohorts are pooled in Fig. 9. The changes are significant both for upright thalli and filamentous holdfasts (Kruskal–Wallis test, *p* < 0.001).

The increase of biomass of upright thalli was significant in early winter, i.e., December to January (Games–Howell test, *p* < 0.05). Peak of the mean biomass of thalli was observed in March, though variations from February to June were not statistically significant. The decrease of thallus biomass was significant from June to July, when thalli became senescent (Games–Howell test, *p* < 0.05).

The results for the seasonal changes in biomass of filamentous holdfasts were similar with the upright thalli. Though the biomass endured until July, it decreased rapidly in August and September (Fig. 9).

**Fig. 10:**
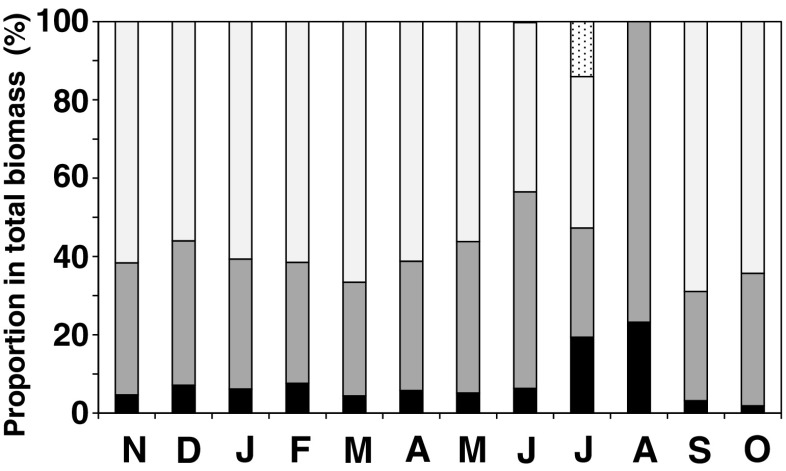
Proportion of each part of *S. fusiforme* in the total biomass. Filamentous holdfasts (*black bars*), axis and branches (both of main and lateral branches; *gray bars*), leaves and vesicles (*white bars*), and receptacles (*dotted bars*)

The proportion of four parts (filamentous holdfasts, branches and axis, leaves and vesicles, receptacles) to the total biomass was shown in Fig. 10. During the growth season (autumn to spring), the leaves and vesicles accounted for ca. 60 % of the total biomass. Only in June and July, reproductive organs (receptacles) accounted for 0.2 and 14 %, respectively. In August, when all thalli were recruits, it was very difficult to identify their axis because they were too short (in case of genets) or difficult to discriminate from filamentous holdfast where they derived (in case of ramets). Therefore, the biomass in August was depicted to be dominated only by leaves and holdfasts.

### Seasonal change of an index of thallus size inequality of the population

**Fig. 11:**
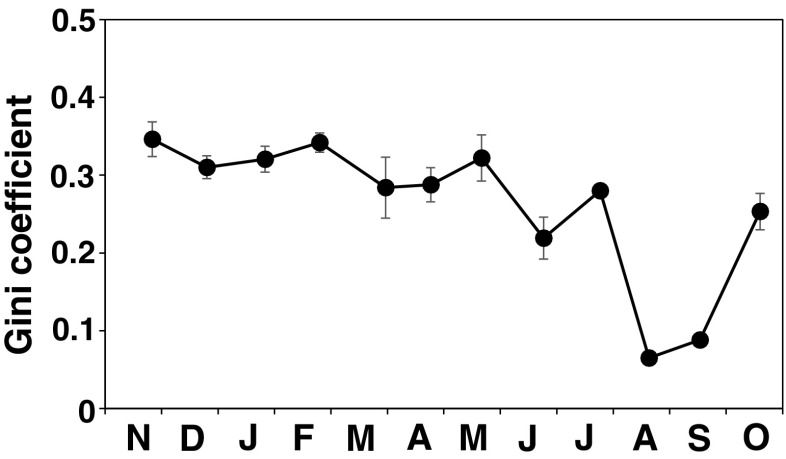
Seasonal fluctuations in the Gini coefficient of the *S. fusiforme* population. *Error bars* indicate standard errors. Thalli of all quadrats were pooled for the calculation for the July, August, and September samples, so *error bars* are not shown

Seasonal fluctuations in the Gini coefficient for the thallus length measurements (Fig. 11) were significant (Kruskal–Wallis test, *p* < 0.05), but a post hoc test failed to detect significant differences between every pair of months. The Gini coefficient was almost constant around 0.3 from November to May in the cohort 2009 and it dropped to 0.21–0.27 in June and July. Though they were not included in the statistical analysis, Gini coefficient values were lower than 0.1 in August and September when all thalli in the population were recruits and young. Gini coefficient values became higher again in October indicating that inequality in size within the population increased during this transition period from recruits to upright thalli.

## Discussion

The seasonal peak of biomass, thallus size, and sexual reproduction of *S. fusiforme* at Zushi-ga-hana occurred in spring to early summer, respectively, and the growth of new upright thalli began in early autumn. This seasonal life cycle is similar with those reported from other habitats along the Pacific coast of Japan (Katada 1940; Suto 1951; Terawaki 1985; Suwa 2014).

It has been reported that vegetative reproduction, rather than sexual reproduction, is the main form of population maintenance of *S. fusiforme* (Arai and Arai 1983). In this study, recruits first appeared in May before receptacles were formed in June and July. Recruitment peaked in August and all recruits developed into upright thalli by October. No new recruits were observed after that. It is also reported that there is a time-lag in the development between recruits of ramets and genets in a *S. fusiforme* population in the Pacific coastal region of Japan, in which young plants of genets first appeared in late autumn in contrast to the first appearance of ramets in May (Suto 1951). As the seasonal population development of *S. fusiforme* in our study was similar to the populations in the Pacific region, the recruits we observed were considered to be ramets and they mainly contributed to maintain the population as the previous studies have indicated (Suto 1951; Arai and Arai 1983). Biomass allocation of receptacles relative to the total biomass at the seasonal peak of maturation was lower in *S. fusiforme* in this study (14 %) than those in other *Sargassum* species reported so far, e.g., 20–24 % in *Sargassum muticum* (Yendo) Fensholt (Arenas and Fernández 1998) and 34 % in *Sargassum macrocarpum* C. Agardh (Murase et al. 2000), also indicating the relative importance of vegetative reproduction in *S. fusiforme*.

Sexual reproduction of *S. fusiforme* has been reported to be induced by the seasonal change in daylength (Park et al. 1995) or by specific accumulation of energy (heat unit) (Zou et al. 2006) as reported in some other *Sargassum* species (Prince and O’Neal 1979; Deysher 1984; Uchida et al. 1991; Uchida 1993; Hwang and Dring 2002). However, environmental factors which induce vegetative reproduction of this species remain unknown. The first appearance of recruits in May, when a rapid increase in water temperature over 15 °C occurred (Fig. 3), indicated that the temperature condition is an important factor. Also, availability of light is considered to be important, as the appearance of recruits peaked in summer after canopy of their previous generation was lost. After the vegetative reproduction, the old filamentous holdfasts of the parent thalli withered, which was reflected in a decrease in its biomass in summer. The lack of recruitment after September could be explained by an inhibitive effect of the developing canopy of new upright thalli on the growth of recruits (possibly, genets), and immature situation of their new filamentous holdfasts which began to be formed in autumn. Some reports mention that the filamentous holdfasts of *S. fusiforme* are perennial (Zou et al. 2006), but in our study, most of the filamentous holdfasts were lost after vegetative reproduction indicating that the holdfasts also have an annual characteristic similar to the upright thalli.

Thalli started upright growth and the biomass of the stand began to increase in early autumn. During this season, a significant decrease in thallus density was also observed. This phenomenon is identical to self-thinning, which is a density dependent or competitively induced mortality occurring in even-aged, monospecific crowded stands of a wide variety of plants including macroalgae (Santos 1995; Scrosati and DeWreede 1997; Creed et al. 1998; Arenas and Fernández 2000; Scrosati 2005). Commonly, in the process of plant self-thinning, a size hierarchy consisting of a few large individuals and numerous smaller ones develops in populations before the occurrence of self-thinning (Arenas and Fernández 2000; Rivera and Scrosati 2008). The small individuals progressively die as a result of asymmetric competition with larger individuals, i.e., typically competition for essential resources for growth such as light or nutrients (Ang 1991; Creed et al. 1996, 1997; Steen and Scrosati 2004). In this study, competition among *S. fusiforme* thalli was considered to intensify due to crowding after the start of elongation of the main branches. The intensified competition caused the development of a hierarchy in thallus size and, probably, mortality of small suppressed thalli which was reflected in the density decrease in early autumn.

During the winter months, however, *S. fusiforme* in this study did not follow the expected patterns of the population dynamics characterized by self-thinning. Thalli continued to grow, and biomass increased gradually until spring. Throughout these seasons, thallus density remained at almost a constant level of about 2500 thalli m^−2^ and there was no sign of mass mortality of the thalli. This is quite different with the self-thinned populations commonly observed in large brown algae such as Fucales and Laminariales, in which the plant density decreases with biomass accumulation during their growth (Creed et al. 1998; Arenas and Fernández 2000; Rivera and Scrosati 2006, 2008).

During the seasonal fluctuation of macroalgal populations, the Gini coefficient varies greatly with the occurrence of recruitment, size hierarchy development, and mortality of individuals (e.g., Ang and DeWreede 1992; Santos 1995; Creed et al. 1998; Arenas and Fernández 2000; Rivera and Scrosati 2008). For other *Sargassum* species in which self-thinning occurs, the Gini coefficient values fluctuated largely with the seasonal development of the population, e.g., from 0.32 to 0.75 in a *S. muticum* population at Cape Penās, Spain (Arenas and Fernández 2000), and from below 0.20 to 0.54 in a *Sargassum lapazeanum* Setchell & Gardner population at the Gulf of California, Mexico (Rivera and Scrosati 2006). Gini coefficient values of *S. fusiforme* for most of the growth season indicate that development of the size hierarchy and mortality was more moderate than in the former two species. Relatively even size distribution of *S. fusiforme* in this study was quite different with a highly skewed size distribution of self-thinned macroalgal populations just before self-thinning occurs. Also, survival probability of *S. fusiforme* thalli was high regardless of their thallus size, at least after the mortality in the early growth phase in early autumn. All these characteristics of *S. fusiforme* cause the Gini coefficient values to be relatively low and stable throughout most of the growth season.

Formation of the main branches of *S. fusiforme* was active in early autumn (September and October), and as in the case of thallus length, a “hierarchy” in the number of main branches which thalli had already appeared in October. After that, however, new formation or turnover of main branches occurred less frequently in winter and spring as mean numbers of main branches per thallus and the frequency of numbers of branches did not change dramatically. This indicates that the density of main branches in the population was almost constant during most of the growth season, which was similar with the seasonal fluctuation of thalli. In some tropical *Sargassum* species (Ateweberhan et al. 2005, 2008), initiation of new primary laterals, or modules, is density-regulated at an individual thallus level to avoid over-crowding. For these species, as thallus density varied little seasonally, no intraspecific competition at the thallus and module level was supposed to exist (Ateweberhan et al. 2005, 2008). In the case of *S. fusiforme* in our study, intraspecific competition occurs in the early phase of the population development, but after that, the survived thalli and main branches coexisted and persisted till the peak of the stand. During this period, each thallus and main branch continued to grow giving the stand a densely-packed feature while maintaining the high density.

The advantages of high density in a macroalgal stand in the intertidal zone are to avoid serious stress by desiccation and high temperature during the low tides (Ang and DeWreede 1992; Scrosati 1996, 2000). Further, high density could provide greater protection from physical forces by dissipating those forces imposed by waves (Scrosati 1996; Nishihara et al. 2011). In Hiroshima Bay, northern seasonal winds prevail almost throughout the whole year except for the summer, and the habitat of *S. fusiforme* in this study, located on the northern coast of Suo-Oshima Is., is frequently exposed to wind waves (Takaya et al. 2005). Protruding parts of larger thalli or branches are easily broken off due to the dragging effect of waves, but the densely packed body of the stand could be physically protected. The loss of the larger parts of the population would be advantageous to smaller thalli by reducing asymmetric competition (Santos 1995). Furthermore, sways of thalli under water motion induced by waves provide sun flecks and nutrients to the under-canopy (Scrosati 2000) which favor the survival and growth of smaller thalli. Although too severe wave conditions prevent formation of *S. fusiforme* stands (Katada 1940), all those physical conditions in our study site are moderate for *S. fusiforme* and can reduce intraspecific competition supporting thalli of various size classes in the population.

Whether the features of *S. fusiforme* observed in this study are specific to the species as a whole or to the studied population remains to be confirmed. However, the thallus density and mean size of *S. fusiforme* at the stand peak reported so far are 1700–2600 thalli m^−2^ and 20–80 cm in the temperate regions in Japan (Katada 1940; Suto 1951; Terawaki 1985; Yotsui et al. 1996; Tanada et al. 2003; Ito et al. 2009a; Suwa, 2014). Thallus density of about 2500 thalli m^−2^ and mean thallus length of 57.4 cm at the peak of the *S. fusiforme* population in our study are similar with these values. Also, harvest biomass has been reported to be 5–20 kg wet weight (w.w.) m^−2^ in those previous reports, and assuming dry biomass accounted for ca. 15 % of wet biomass (Ofusa 2011), the peak biomass observed in our study was >20 kg w.w. m^−2^. Therefore, the *S. fusiforme* population in our study exhibited productivity close to the highest values reported in temperate Japan. The high productivity observed is attributable to the densely-packed biomass per unit of volume of the population with a feature of high density. The feature is considered to be supported in its development by a physical condition, i.e., the advantages of moderate wave effects.

It has been reported that habitats of *S. fusiforme* in Asia are moderately exposed to waves (Katada 1940; Tanada et al. 2003; Zou et al. 2006). In one of the oldest ecological studies on *S. fusiforme*, a positive relationship between thallus density and biomass of *S. fusiforme* stands in the harvest season was observed in the Boso Peninsula on the Pacific region in Japan (Katada 1940). From the results of our study, it is suggested that the positive relationship between density and biomass would have developed along the gradients of physical conditions at the habitats, which could affect the carrying capacity of *S. fusiforme* stands. Therefore, evaluations of the physical conditions eco-physiologically favored for *S. fusiforme* are needed to establish effective techniques to enhance natural resource of *S. fusiforme.*
